# In-situ upgrading of Egyptian heavy crude oil using matrix polymer carboxyl methyl cellulose/silicate graphene oxide nanocomposites

**DOI:** 10.1038/s41598-024-70843-3

**Published:** 2024-09-09

**Authors:** Eman M. Mostafa, Alaa Ghanem, Rasha Hosny, Raghda El-Nagar

**Affiliations:** 1 PVT Lab., Production Department, Egyptian Petroleum Research Institute, Nasr City, Cairo, 11727 Egypt; 2 Faculty of Postgraduate for Nanotechnology, Cairo University, El-Sheikh Zayed, 12588 Egypt; 3 PVT Services Center, Egyptian Petroleum Research Institute, Nasr City, Cairo, 11727 Egypt; 4 EOR Lab., Production Department, Egyptian Petroleum Research Institute (EPRI), 1 Ahmed El-Zomor St., Nasr City, Cairo, 11727 Egypt; 5 Petroleum Testing Lab, Analysis and Evaluation Department, Egyptian Petroleum Research Institute, Nasr City, Cairo, 11727 Egypt

**Keywords:** Catalytic aquathermolysis, Heavy crude oil, Increase heavy oil productivity, Matrix polymer, Nanocomposites, Chemistry, Fossil fuels

## Abstract

This study delves into catalytic aquathermolysis to enhance the economic viability of heavy oil production by in-situ upgrading technique. It is known that introducing nanocatalysts would promote the aquathermolysis reaction. Therefore, in this study, the effect of matrix polymer carboxyl methyl cellulose/silicate graphene oxide nanocomposites (CSG1 and CSG2) in the catalytic aquathermolysis of Egyptian heavy crude oil was studied. Characterization techniques including Fourier-transform infrared (FTIR), X-ray diffraction (XRD), Dynamic light scattering (DLS), Brunauer–Emmett–Teller (BET) surface area analysis, Scanning electron microscopy (SEM), and thermogravimetric analysis (TGA) were used to evaluate the structure of the synthesized nanocomposites. Results reveal CSG2 has higher crystallinity and superior dispersion compared to CSG1, and both exhibited a good stability in aqueous suspensions. CSG2 enriched with graphene oxide, demonstrates superior thermal stability, suitable for high-temperature applications such as catalytic aquathermolysis process. Single factor and orthogonal tests were used to assess the catalytic aquathermolysis performance of the prepared nanoparticles. The obtained results revealed that the optimum conditions to use CSG1 and CSG2 are 40% water concentration, 225 °C temperature, and 0.5 wt% catalyst percentage. Where, CSG2 showed better viscosity reduction (82%) compared to CSG1 (62%), highlighting its superior performance in reducing the viscosity of heavy oil. Numerical results from SARA analysis, gas chromatography, and rheological testing confirmed the catalytic aquathermolysis's efficacy in targeting asphaltene macromolecules and producing lighter hydrocarbon fractions.

## Introduction

The exploitation of heavy and extra-heavy oil resources is a critical focus in the global energy sector due to their abundance and potential to supplement depleting conventional oil reserves^1^. Heavy crude oil is distinguished by its high viscosity and density, as well as its large and complex hydrocarbon molecules^2^. Extra-heavy oil, even more viscous and dense, often requires advanced extraction and processing techniques^3^. The global energy crisis, driven by population growth, industrialization, and urbanization, underscores the need to explore alternative energy sources as conventional oil reserves deplete^4,5^.

Heavy and extra-heavy oil reserves are abundant in regions such as Venezuela, Canada, and parts of the Middle East, representing a significant portion of the world's oil reserves^6^. However, the extraction and refining of these oils pose considerable challenges due to their high viscosity^7,8^. Traditional developing methods are often inadequate, prompting the development of specialized techniques like thermal recovery methods^9,10^. These include Steam Assisted Gravity Drainage (SAGD), widely used in Canada’s oil sands, and Cyclic Steam Stimulation (CSS), which employ steam to reduce oil viscosity and enhance mobility^11^. In-situ combustion and electric heating methods further aid in extracting these challenging resources^12,13^. Despite these technological advancements, the environmental implications of extracting and refining heavy and extra-heavy oils cannot be overlooked^14^. The processes often result in higher greenhouse gas emissions and environmental degradation, prompting the need for cleaner and more sustainable methods^15^​. One promising approach to improving the extraction and processing of heavy crude oil is aquathermolysis, which upgrades heavy oil into more marketable products through heat and water^16^. Aquathermolysis, developed by Hyne et al.^17^, breaks down heavy hydrocarbons, improves the quality and market value of the extracted oil. While traditional methods for reducing viscosity often focus on manipulating temperature, which can be technically and economically challenging^18^. Catalytic aquathermolysis, a variation of this process, incorporates catalysts to enhance reaction efficiency and product selectivity, operating at lower temperatures and pressures to reduce energy consumption and operational costs^19,20^.

Several studies have demonstrated that the incorporation of catalysts in the aqueous phase can significantly reduce oil viscosity during the aquathermolysis process^21–25^. This enhanced oil mobility is likely due to a decrease in the content of high-viscosity components like resins and asphaltenes^26–29^. Wang et al. showed that adding an acidic catalyst, like aromatic sulfonic iron, triggers a series of chemical reactions that break down resins and asphaltenes. These reactions involve cleaving C–C bonds, C=C bonds, conjugated structures, and C–X bonds (where X represents heteroatoms). This breakdown process produces low-molecular-weight hydrocarbons that dissolve in the oil, leading to a net decrease in oil viscosity^30^. These findings are corroborated by similar mechanisms reported in other studies^31–36^. Wen et al.^37^ investigated the effectiveness of H_4_SiW_12_O_40_ in reducing the viscosity of heavy oil from the Shengli reservoirs. They observed a significant viscosity reduction exceeding 67% after a 36-h reaction at 240 °C. In 2009, Chen et al. employed amphiphilic catalysts, combining aromatic sulfonic acid anions with iron cations, to achieve a remarkable 90.7% reduction in the apparent viscosity of extra-heavy oil at 200 °C. This significant improvement is attributed to the catalyst's stability at the water–oil interface.

Building on these advancements, this paper explores the synthesis and characterization of two Carboxyl Methyl Cellulose/Silicate Graphene Oxide Nanocomposites with 10% and 20% CMC, named CSG1 and CSG2 respectively. The prepared materials have undergone comprehensive characterization through various analytical techniques, including FTIR, XRD, DLS Surface area (BET), SEM and TGA. Many catalysts fail to maintain stability at high temperatures, which is necessary for processes like catalytic aquathermolysis. The superior thermal stability of the prepared nanocomposites address this issue, making them viable catalysts for high-temperature applications. To the best of our knowledge, the synthesized materials were investigated for the first time as promising catalysts to in-situ upgrade the heavy crude oil using the catalytic aquathermolysis process. Moreover, the effect of the prepared materials on the rheological properties of the crude oil was examined. In addition, the change in the compositional analysis of the crude oil before and after the catalytic aquathermolysis was investigated.

## Materials and experimental

### Materials

The chemicals used in this study include Sodium silicate solution (Na₂SiO₃, 99%), sodium nitrate (NaNO3), n-heptane(99.7%), toluene (99.5%), benzene (99%), sulfuric acid (H2SO4, 96.90%), distal water, deionized (DI), potassium permanganate (KMnO4), hydrogen peroxide (H_2_O_2_, 30%), hydrochloric acid (HCl, 37%) and ethanol (99.9%). All chemicals were purchased from Sigma- Aldrich, and were used without any further purification. Carboxy methyl cellulose Sodium salt (250,000 g/mol) was purchased from ADWIC.

### Synthesis of matrix polymer carboxyl methyl cellulose/silicate graphene oxide nanocomposites (CSG1 and CSG2)

**Fig. 1:**
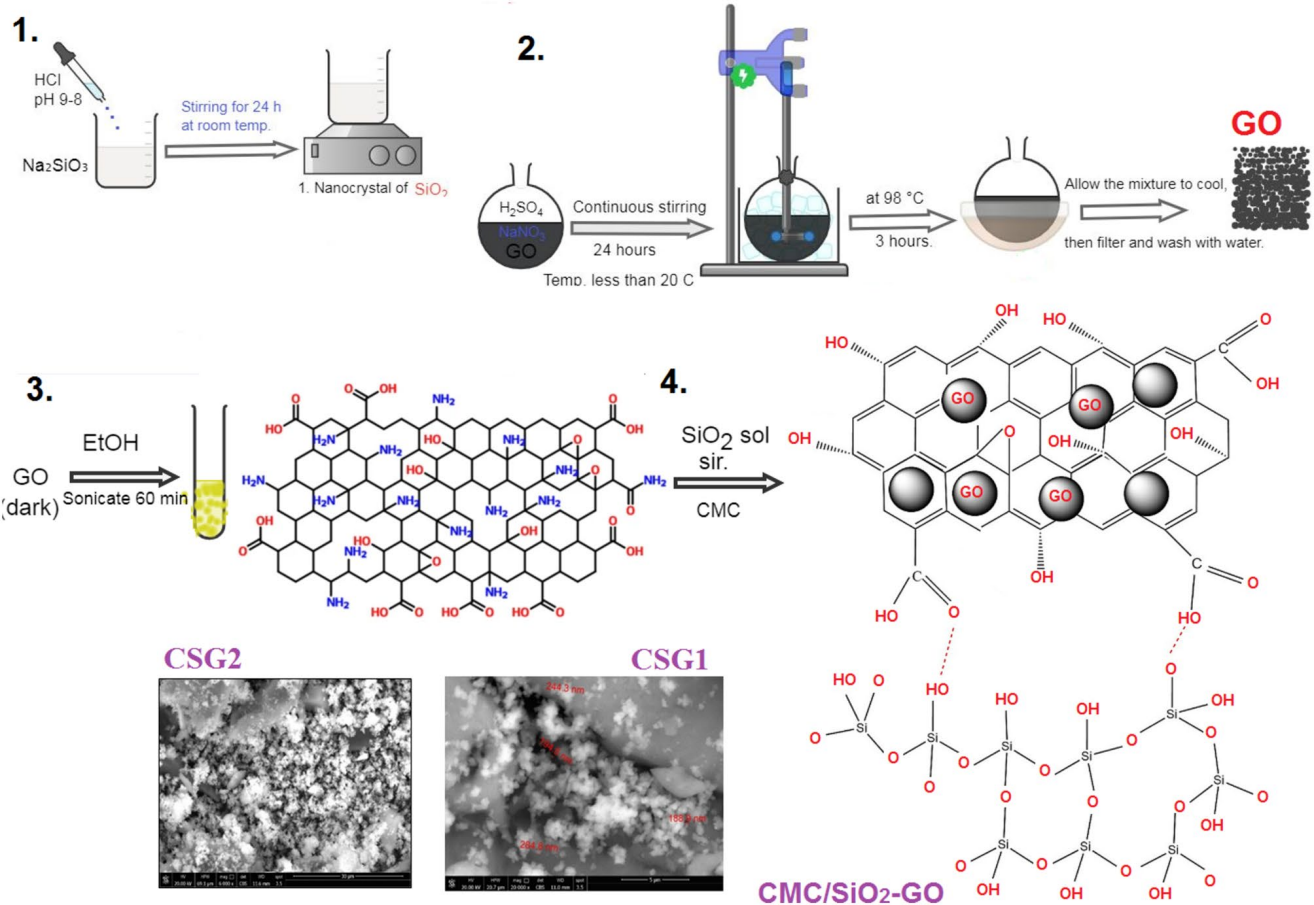
Scheme of the matrix polymer carboxyl methyl cellulose/silicate graphene oxide nanocomposites.

Figure 1 illustrates the synthesis process for matrix polymer carboxymethyl cellulose/silicate graphene oxide nanocomposites (CSG1 and CSG2). To synthesize SiO_2_ nanocrystals, a sodium silicate solution (10gm, (500 ml) was prepared in distilled water, and hydrochloric acid (HCl, 0.1 M) was gradually added until the pH reached 8–9. After 24 h of aging at room temperature, the silica gel was washed and dried at 90 °C for 18 h, yielding nanosilica powder.

For GO synthesis, graphite powder (3 g) and sodium nitrate (1.5 g) were mixed in concentrated sulfuric acid (46 mL, 96.90%) with continuous stirring. Gradual addition of potassium permanganate (9 g) at a temperature below 20 °C was followed by heating at 98 °C for 3 h. The resulting GO was washed with HCl and H_2_O, then subjected to centrifugation, filtration, and drying at 80 °C.

In the synthesis of the SiO-GO nanocomposite, 0.1 g of GO powder was dispersed in ethanol (20 ml) using an ultrasonic bath for 1 h. SiO_2_ sol. (dispersed in ethanol) was added, and the mixture was further ultra-sonicated for 30 min, stirred for 12 h, and subjected to centrifugation, washing with ethanol and deionized water, and drying at 60 °C for 12 h.

### Characterization of CSG1 and CSG2 nanocomposites

Various characterization techniques were employed to analyze the synthesized Matrix Polymer CMC/SiO_2_-GO nanocomposites. FTIR analysis utilized a Nicolet Is-10 FT-IR spectrophotometer with the KBr method; Thermo Fisher Scientific. XRD investigation utilized a Pan Analytical Model X' Pert Pro to understand molecular arrangement X-ray diffraction were recorded using a Pan Analytical Model X' Pert Pro, equipped with CuKα radiation (λ = 0.1542 nm), Ni-filter and general area detector. DLS was carried out using Zeta Sized Nano Series, Nano ZS, Malvern Instruments. Surface area determination utilized using Quantachrome NOVA 3200 automated gas sorption system (USA) at – 196 °C. The surface area was calculated using the Brunauer–Emmett–Teller (BET) equation from the adsorption branch in the relative pressure P/P_o_ range of 0.05–0.35. The pore size distribution was calculated based on desorption branches of the nitrogen isotherms using the Barrett-Joyner-Halenda (BJH) method. SEM was performed on JEOL JEM 3500 electron microscope. TGA was performed using (Shimadzu TGA-50, Japan).

### Laboratory work on catalytic aquathermolysis

In this research, Egyptian heavy oil samples were obtained from Egyptian Petroleum Company, Egypt with the physical properties shown in Table 1.

**Table 1:** physical properties of the heavy crude oil sample.

Item	Method	Value
Kinematic viscosity, cSt	ASTM D 445	423.6 (± 0.2%)
Sulfur content in ppm	ASTM D-4294	15,285 (± 0.05%)
Density, g/cc	ASTM D 4052	0.946 (± 0.002 g/cc)
API°		17.6
Average MWt	…	367

Evaluating the catalytic performance of the prepared catalysts was performed using orthogonal and single factor experiments (Table 2). All the experiments were carried out by adding successfully a designed mass ratio of oil, water and one of the two prepared catalysts to a designed high pressure reactor (Parr, 500 ml). The Parr reactor was pressurized to 20 bar as an initial pressure. During the cracking experiments, the amount of the produced gases increased and therefore the reaction pressure increased to about 50–60 bar. After the experiments were finished and the reactor temperature was cooled to room temperature, the treated oil was taken to be evaluated via viscosity measurements according to ASTM D445 with an uncertainty of ± 2%, using SVM 3001 viscometer. The rate of viscosity reduction (∆ɳ) was calculated for each experiment using the following equation:

$$ \Delta \eta \, \% = \left( {\left( {\eta_{{\text{o}}} - \eta } \right)/\eta_{{\text{o}}} } \right) \times {1}00 $$

where, ∆η is the rate of viscosity reduction, η_o_ is the viscosity of the untreated crude oil and η is the viscosity of the treated oil.

**Table 2:** The proposed single factors of the aquathermolysis experiments.

Item	Water concentration, (v/v %)	Temperature (°C)	Catalyst (wt%)
1	20	75	0.1
2	30	150	0.5
3	40	225	1.00

The compositional analyses of the produced oils from the catalytic aquathermolysis reaction were identified on a Gas Chromatography-Agilent Technologies, while the sulfur content was measured on sulfur analyzer (Spectroscan S-SL).

#### Group composition analysis (SARA)

SARA analysis (saturates, aromatics, resin, and asphaltene) was performed to study the oil’s group composition, where asphaltene content was determined and isolated first. The asphaltene utilized in our study was extracted using a precipitation method with excess of n-heptane according to IP-143^38^. Then, maltene (deasphated oil) was separated via liquid adsorption chromatography using a specialized glass column (20 × 500 mm) filled with 0.04–0.16 mm aluminum oxide adsorbent was used for this separation^38^. The column was pre-wetted with 50 ml of n-hexane before introducing the maltene dissolved in n-hexane. Proceeding with meticulous care, the column was subjected to sequential elutions. Specifically, it was sequentially eluted with 200 ml of n-hexane, 200 ml of Benzene, and 200 ml of a toluene/ethanol mixture in a ratio of 3:1 volume %. This strategic sequencing was employed to facilitate the systematic elution of distinct components: saturated hydrocarbons, aromatic compounds, and resins, respectively.

#### Rheological study

In this study, the rheological properties of the treated and untreated crude oil were investigated using the Anton Paar MCR102 rheometer, in which the flow curves were recorded at shear rates between 10^–1^ and 10^3^ s^−1^. Moreover, a viscoelastic study was performed to measure the effect of catalytic aquathermolysis on the treated crude oil. The measurements were conducted at two degrees of temperature: 30 and 50 °C. Prior to the test, all samples underwent a 5-min equilibration period at the designed measurement temperature. After that, the rheometer underwent a brief calibration routine involving taking the average of three readings for each test to verify its repeatability.

## Results and discussion

### Characterizations

#### FTIR

**Fig. 2:**
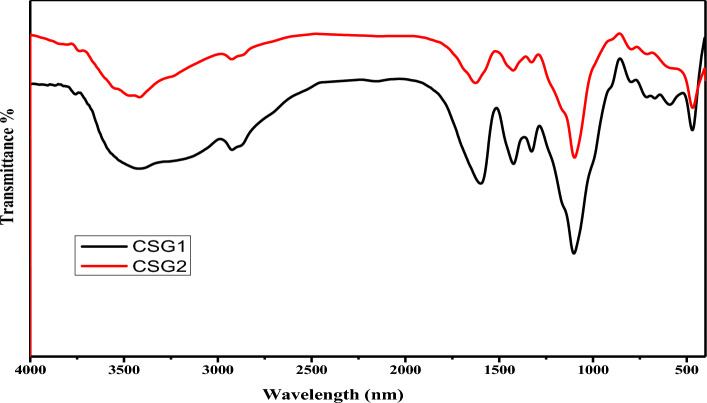
FTIR spectra of the matrix polymer of CSG1 and CSG2 nanocomposites.

Figure 2 shows the FTIR spectra of the CSG1 and CSG2 nanocomposites reveal commonalities and differences in their functional groups. Both nanocomposites exhibit broad peaks between 3000 and 2500 cm^−1^, indicating the presence of O–H stretching vibrations and hydroxyl groups. Additionally, a peak at around 1600 cm^−1^ indicates the presence of C=O stretching vibrations and carbonyl groups in both nanocomposites. The distinguishing feature is a peak at approximately 1500 cm^−1^ in the spectrum of both CSG1 and CSG2, attributed to N–H bending vibrations.

#### XRD

**Fig. 3:**
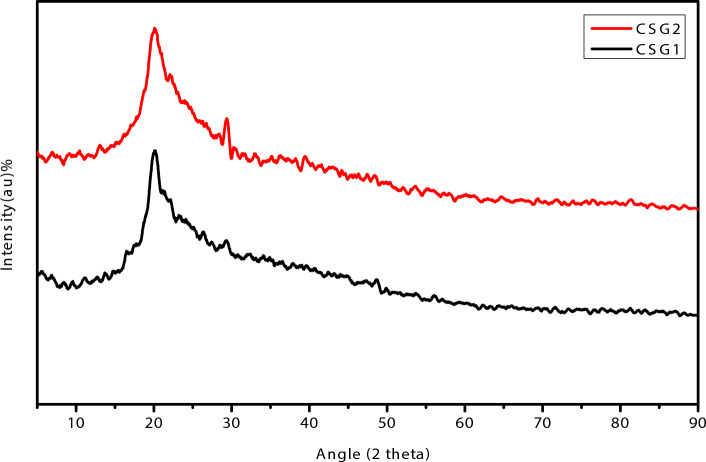
X-ray diffraction (XRD) pattern of matrix polymer of CGS1 and CGS2 nanocomposites.

Figure 3 described the X-ray diffraction (XRD) pattern of two materials, CSG1 and CSG2. The XRD pattern is a plot of the intensity of X-rays scattered by a material as a function of the angle of the scattered X-rays. The peaks in the pattern correspond to the spacing between atoms in the material. The XRD pattern for CSG1 and CSG2 shows that they have a similar crystalline structure. The peaks in the pattern are at the same angles for both materials, which suggest that the atoms are arranged in the same way in both materials. However, the intensity of the peaks is different for the two materials. This indicates that the materials have different crystal sizes or that there is a different amount of material in each sample. The XRD peaks for CSG2 are higher than the intensity of the peaks for CSG1. This suggests that CSG2 is more crystalline than CSG1. The higher crystallinity of CSG2 may be due to the higher concentration of graphene oxide in the nanocomposite. The XRD peaks for CSG1 and CSG2 can be assigned to the peak at around 2θ = 10° can be assigned to the (002) plane of CMCNa. The peak at around 2θ = 20° can be assigned to the (001) plane of SiO_2_. The peak at around 2θ = 40° can be assigned to the (002) plane of graphene oxide. The presence of these peaks indicates that the CMCNa, SiO_2_, and graphene oxide are well-dispersed in the nanocomposite. The XRD results illustrated that CSG2 is a more crystalline material than CSG1. This may be due to the higher concentration of graphene oxide in the nanocomposite. The higher crystallinity of CSG2 may lead to improved mechanical properties. The Scherer equation, Eq. (1)^39^, was used to estimate the mean particle size of the produced nano catalyst CSG1 and CSG2 was approximately 70.01 nm and 21.38 nm respectively.

1 $$ {\text{D}} = \frac{0.89\lambda }{{\beta \cos \theta }} $$

where, D is the mean crystallite size, β is the broadening of the diffraction line measured at half-maximum intensity, λ is the wavelength of the X-ray radiation, and θ is the Bragg angle.

#### BET, DLS and zeta potential

**Fig. 4:**
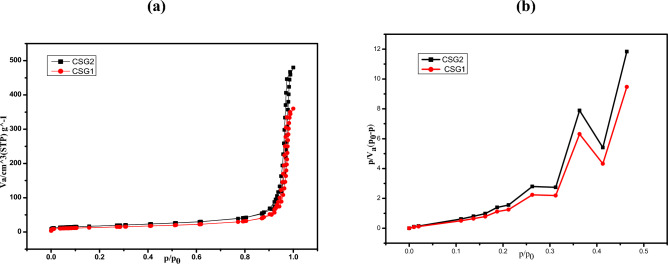
The isotherms (**a**) and pore size distribution (**b**) of matrix polymer of CSG1 and CSG2 nanocomposites.

Figure 4 displays the adsorption–desorption isotherms of N_2_ of matrix polymer of CSG1 and CSG2 nanocomposites. The analysis of the porosity of the matrix polymer of CSG1 and CSG2 composites via adsorption–desorption isotherms of N_2_ at 273 K revealed specific surface areas of 0.577312 m^2^/g and 0.72164 m^2^/g, respectively, calculated using the Brunauer–Emmett–Teller (BET) model. Additionally, the average pore size diameter, determined by the Barrett-Joyner-Halenda (BJH) model, was found to be 46.963 nm and 58.704 nm for CSG1 and CSG2, respectively (Table 3).

**Table 3:** BET/Z-average/zeta potential for matrix polymer CSG1 and CSG2 nanocomposites.

BET/Z-average/Zeta potential	CSG1	CSG2
V_m_ [cm^3^(STP) g^−1^]	0.13264	0.1658
a_s,BET_ [m^2^ g^−1^]	0.577312	0.72164
Total pore volume [cm^3^ g^−1^]	0.008473	0.010591
Average pore diameter[nm]	46.9632	58.704
Z-average (nm)	459.1	485.15
Zeta potential (mV)	− 61.7	− 64.1

**Fig. 5:**
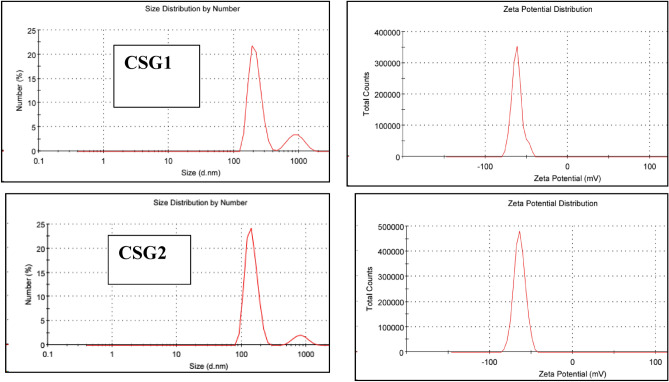
Size distribution and zeta potential of matrix polymer of CSG1 and CSG2 nanocomposites.

Furthermore, measurements of Z-Average and Zeta potential in aqueous solutions, depicted in Fig. 5, provided valuable insights into the dispersion stability of colloids and the electrostatic interaction between different graphene oxide sheets. The results showed Z-Average values of 459.1 nm and 485.15 nm, and Zeta potential values of − 61.7 mV and − 64.1 mV for CSG1 and CSG2 nanocomposites, respectively. These highly negative values indicate that the nanocomposites are stable in aqueous suspensions due to strong electrostatic repulsion resulting from the presence of oxygen species on their surfaces. According to literature, a zeta potential absolute value higher than 30 mV indicates the stability of graphene oxide compound suspensions^40^. From these data, it was evident that the CSG1 and CSG2 nanocomposites possess high specific surface areas and large pore size diameters. Additionally, their stability in aqueous suspensions due to their negative zeta potential makes them promising candidates for various applications.

#### Morphological analysis

**Fig. 6:**
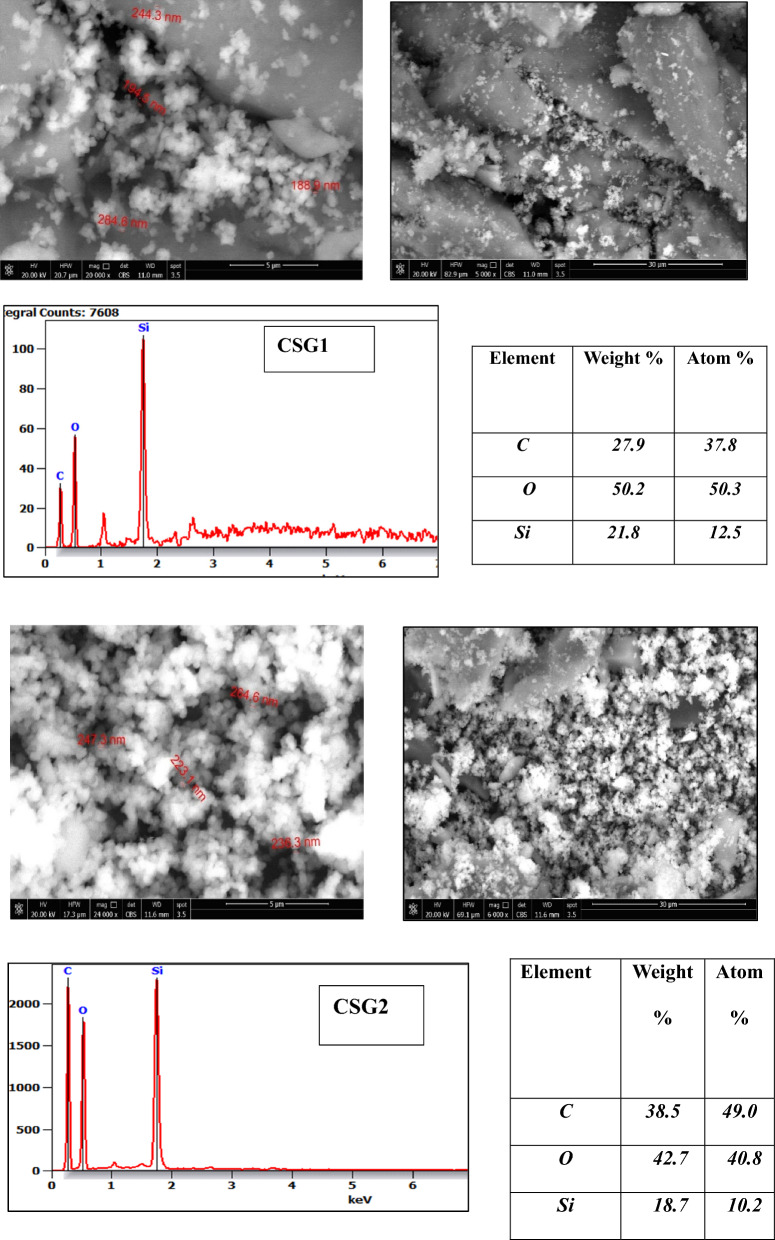
SEM and EdX of matrix polymer of CSG1 and CSG2 nanocomposites.

Figure 6 utilizing the Scanning Electron Microscope (SEM), the morphologies of CSG1 and CSG2 nanocomposite matrix polymers. In Fig. 6a,b, the presented graphene oxide (GO) displays a carpet-like pattern, likely attributed to residual bound moisture and the presence of hydroxyl, epoxy, and carboxyl functional groups on the GO surface^41^. The graphene sheets exhibit intrinsic microscopic roughening and out-of-plane deformations (wrinkles), with some dispersed GO sheets connecting randomly to form a porous structure with numerous cavities or holes. This results in a cross-linked flaky structure with excellent dispersibility and an average size perspective of approximately (188–284 nm) and (223–264 nm) for composite matrix polymers CSG1 and CSG2, respectively.

SEM images of the CSG2 nanocomposite samples reveal superior dispersion, indicating a larger specific surface area that promotes enhanced catalytic activity. Additionally, Energy Dispersive X-ray (EDX) measurements were conducted for validation (Fig. 6). The CSG2 nanocomposite sample prominently exhibits C, O, and Si elements. Conversely, the expected C, O, and Si element peaks in the structure were confirmed by EDX investigation, providing evidence for the uniform distribution of C, O, and Si particles within the hybrid SiO_2_-GO-CMC nanocomposite material, forming SiO_2_-GO nanohybrids between the precursor and GO. The C, O, and Si content in the CSG1 EDX is higher than in CSG2 because EDX measures only the elements present on or near the surface. Therefore, the analysis may not fully reflect the overall composition of the samples. This can result in discrepancies between the surface layers and the bulk of the sample. Additionally, the distribution of nano silica in the sample may not have been homogeneous.

#### Thermogravimetric analysis (TGA) of CSG1 and CSG2

**Fig. 7:**
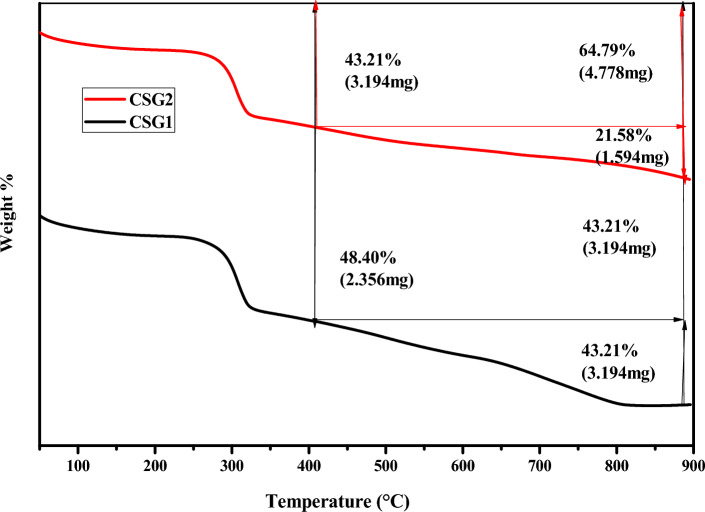
TGA analysis for matrix polymer of CSG1 and CSG2 nanocomposites.

Figure 7 shows the TGA analysis and the thermal stability analysis of SiO_2_-GO/CMC nanocomposites. The TGA curves for SiO_2_-GO/CMC nanocomposites (CSG1 and CSG2) reveal a two-stage decomposition process. Moisture evaporates occurs around 100 °C, followed by the second stage involving organic component decomposition, which starts at 200 °C for the 10% composite and 250 °C for the CSG2composite. The CSG1 composite decomposes more rapidly than the CSG2, indicating higher thermal stability for the latter. At 900 °C, the CSG2 sample retains 56.79% weight, while the CSG1 sample retains 35.21%. This signifies greater thermal stability for the CSG2 composite due to lower organic content. Specific observations include moisture loss, GO surface decomposition (300–500 °C), CMC matrix decomposition (500–700 °C), and SiO_2_ nanoparticle decomposition (> 700 °C). The graphene oxide in CSG2 enhances stability, making it suitable for high-temperature applications. Therefore, CSG2, enriched with graphene oxide, is recommended for high-temperature applications due to its superior thermal stability compared to CSG1.

### Catalytic aquathermolysis results

To meet the future energy demands, heavy oil must be progressively upgraded into lighter and more valuable oil. Catalytic aquathermolysis is one of the most promising techniques in upgrading and decreasing the viscosity of the heavy crude oil in the presence of catalyst. The incorporation of nanomaterials as potential catalysts in the aquathermolysis process is vital and very efficient.

#### Effect of CSG1 and CSG2 on the viscosity reduction of heavy oil

The role of CSG1 and CSG2 nanocomposites in the aquathermolysis process was investigated by comparing the viscosity reduction of each run. It is obvious that the orthogonal experiments were based on three factors: water content, temperature, and catalyst percent, as shown in Table 4. The time of each experiment was adjusted at five hours. The data in Table 4 reveals that the optimum conditions for both catalysts are 40%, 225 °C, and 0.5 wt% for the water concentration, temperature, and catalyst percent, respectively. In addition, the viscosity reduction using CSG2 is much better than that of CSG2, where the viscosity reduction reached 62% and 82%, respectively. This indicates that there is a considerable degree of viscosity reduction of the crude oil after using CSG2, even at relatively lower temperatures.

**Table 4:** Designed experiments of catalytic aquathermolysis of the heavy crude oil.

No	Water concentration (v/v %)	Temperature (°C)	Catalyst (wt%)	Viscosity reduction (%)		
				Aquathermolysis	CSG1	CSG2
1	20	75	0.1	7	− 12	11
2	20	150	0.5	12	15	51.5
3	20	225	1.00	− 17	55	69
4	30	75	0.5	5	14	35
5	30	150	1.00	14	59	75
6	30	225	0.1	− 8	51	78
7	40	75	1.00	8	24	33
8	40	150	0.1	14	43	57
9	40	225	0.5	22	62	82
10	20	75	0.5	− 15	7	27
11	20	150	1.00	− 5	44	48
12	20	225	0.1	11	39	52

#### Group composition analysis (SARA)

According to viscosity reduction data, it is clear that the optimal conditions referred to 0.5 wt% of catalyst at 225 °C in presence of 40% water concentration for both catalysts (CSG1 and CSG2). Consequently, the yielded oils under these conditions in the absence and the presence of catalysts were analyzed via SARA technique to study the difference in the group chemical composition. In which, four components were separated from the oil namely saturates, aromatic, resin and asphaltene using the column chromatography. Compared to the crude oil, the catalytic aquathermolysis using both CSG1 and CGS 2 samples show a substantial increase in lighter fractions, with saturates rising by 14.3% and 21.7% and aromatics by 12.8% and 26%, respectively as shown in Table 5. This is accompanied by a decrease in heavier components, with resins dropping by 7.7% and 17.9% and asphaltenes experiencing the most significant decline of 28.3% and 41.6% for CSG1 and CSG2, respectively. These results suggest that the catalytic aquathermolysis primarily targets asphaltene macromolecules, breaking them down into lighter and more desirable hydrocarbon fractions.

**Table 5:** Group compositional analysis of the crude oil, the treated oil via aquathermolysis, and the treated oil via catalytic aquathermolysis using CSG1 and CSG2.

Item	Sulfur content, ppm	Component			
		Saturates, wt%	Aromatics, wt%	Resin, wt%	Asphaltene, wt%
Untreated oil	18,285	24.4	27.3	32.3	15.9
Aquathermolytic oil	17,736	25.7	28.6	31.1	14.5
CSG1	16,134	27.9	30.8	29.8	11.4
CSG2	13.968	29.7	34.4	26.5	9.3

#### FT-IR of the extracted asphaltene

**Fig. 8:**
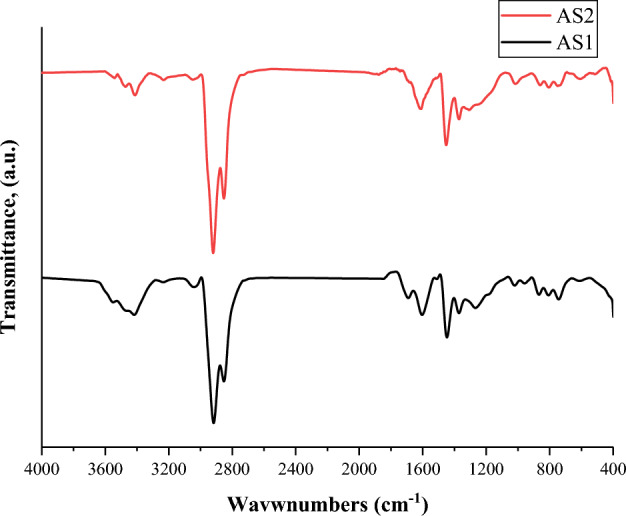
FT-IR of the extracted asphaltene before (AS1) and after (AS2) aquathermolysis process at the optimum conditions.

To investigate the effect of catalytic aquathermolysis on the heavy crude oil, FT-IR spectroscopy was employed. The analysis focused on asphaltene extracted under the optimal experimental conditions and compared it to the original asphaltene before the treatment. As illustrated in Fig. 8, the FT-IR spectra reveal distinct differences between the two samples. Notably, the observed peaks at 3417 and 3412 cm^−1^ are attributed to the O–H stretching vibration, potentially indicating the presence of carboxylic, phenolic, or alcoholic groups within the asphaltene structure. Additionally, the FT-IR spectra showed peaks near 1609 and 1602 cm^−1^ for the untreated (AS1) and the treated (AS2) asphaltene, respectively, which are associated with the stretching vibrations of conjugated polyene C=C bonds and aromatic ketone C=O bonds in asphaltene. This shift towards lower wavenumbers in the treated asphaltene may be indicative of increased condensation of aromatic groups. In the untreated asphaltene, peak at 1712 cm^−1^ was observed, corresponding to aliphatic esters, aromatic esters, aliphatic carboxylic acids, and aromatic carboxylic acids. This peak disappeared in the treated asphaltene, suggesting the breaking of the C–C bond in the long alkyl chain influenced by the catalytic aquathermolysis process.

**Fig. 9:**
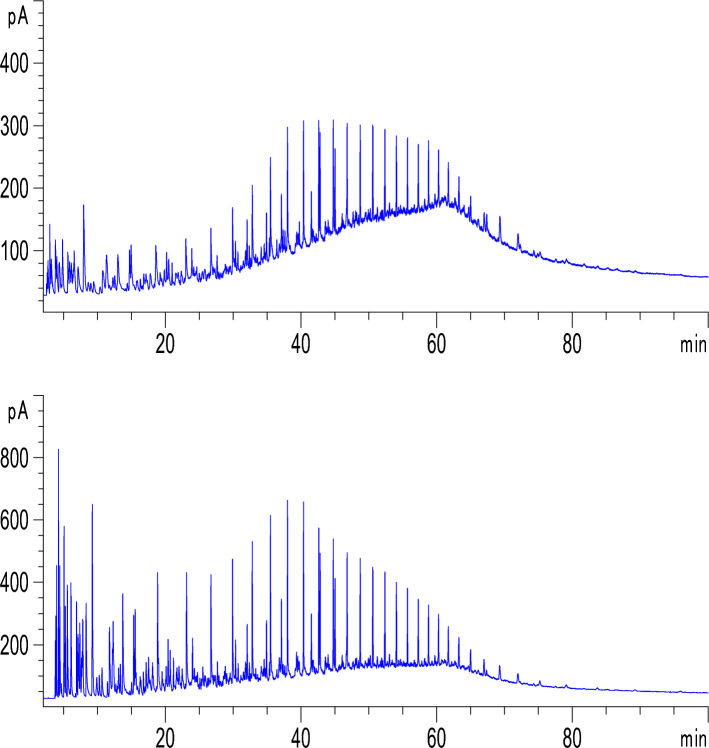
Gas chromatograph for crude oil (**a**) before and (**b**) after catalytic aquathermolysis using CSG2 at the optimum conditions.

**Fig. 10:**
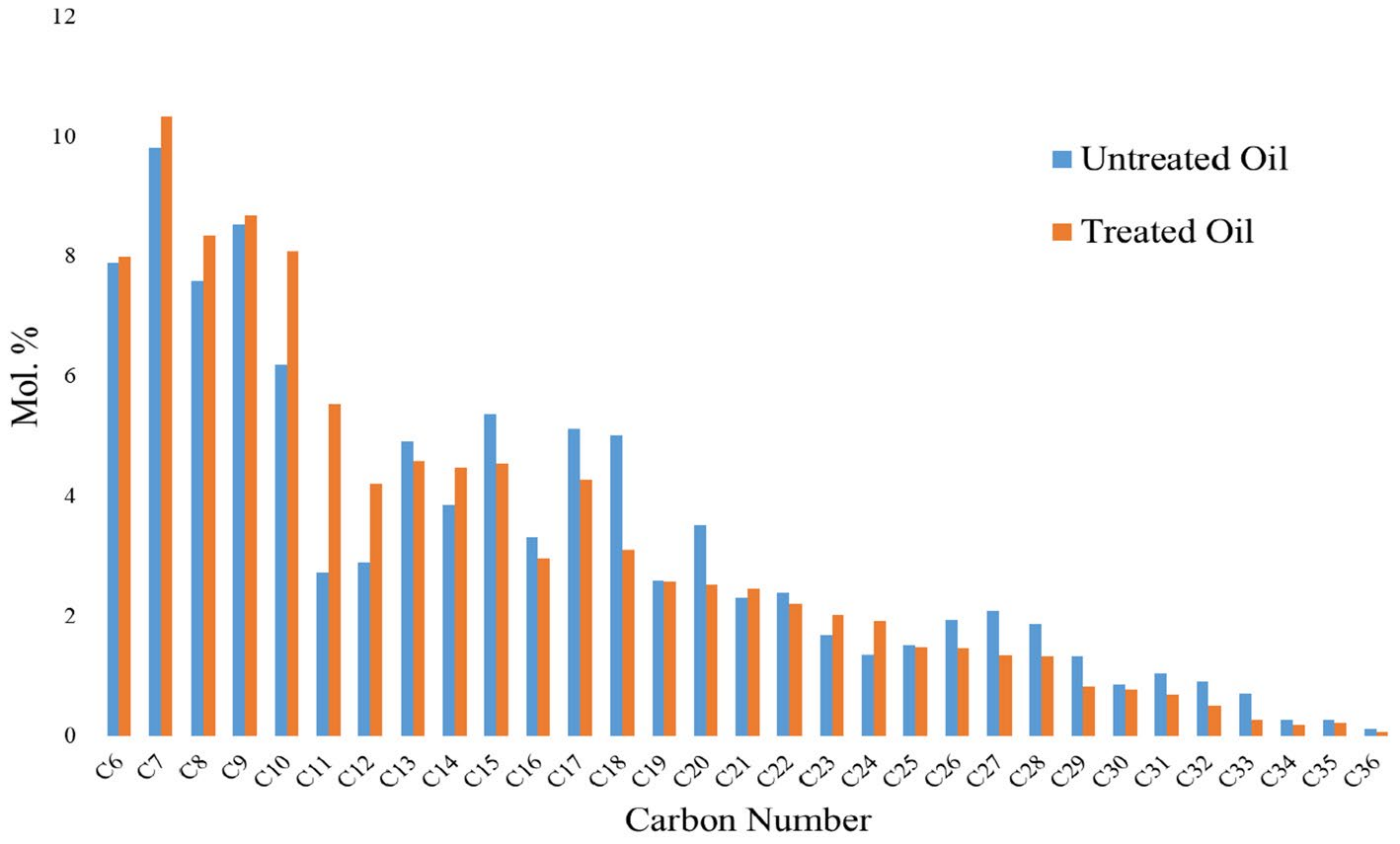
Comparison between the composition analysis of the crude oil before and after catalytic aquathermolysis using CSG2 at the optimum conditions.

Gas chromatography analysis revealed that the composition of crude oil was significantly modified after undergoing catalytic aquathermolysis treatment with CSG2 (Fig. 9a,b). The most notable change was a dramatic increase in the light hydrocarbon fraction (C_6_ to C_12_), with their mole percent rising from 7.8, 9.8, 7.5, 8.5, 6.1, 2.7, and 2.9 to 8.0, 10.3, 8.3, 6.8, 8.0, 5.5, and 4.2 mol% as shown in Fig. 10. This is possibly due to the degradation of alkyl chains in the heavy components like asphaltenes. This is in a good agreement with the reported results obtained by Aliev et al., where they stated that the content of the low molecular weight hydrocarbon was increased during the in-situ catalytic upgrading of heavy crude oil using nickel tallate catalyst^42^. This is also supported by the decrease in mole percent of the heavy components (C_13_ and higher).

### Rheological behavior

**Fig. 11:**
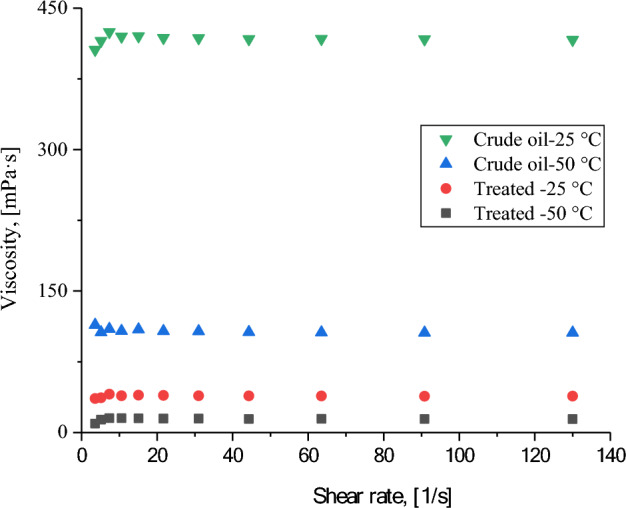
Viscosity flow behavior of crude oil before and after catalytic aquathermolysis using CSG2 at 25 and 50 °C.

Catalytic aquathermolysis aims to improve the flowability of heavy crude oil by reducing its viscosity, making it easier to transport within the reservoir or pipeline. The treated oil sample displayed shear thinning behavior as shown in Fig. 11. As the temperature rose from 25 to 50 °C, its apparent viscosity decreased. This behavior is likely due to the temperature-induced weakening of interactions between the heavy molecules, such as asphaltenes, which tend to clump together and increase viscosity. The results confirm that the treated oil exhibited a significantly lower viscosity compared to the untreated crude oil, where the viscosity of the crude oil at two different temperatures (25 and 50 °C) was decreased remarkably from around 420 and 110 cp to around 40 and 14 cp after using CSG2 at the optimum conditions of the catalytic aquathermolysis process. This is almost attributed to the effect of heat and the used catalysts, where they work together to break down the asphaltene molecules into smaller, lighter molecules. This enhances the overall viscosity reduction of the crude oil.

A crucial tool for studying the viscoelastic behavior of the hydrocarbon liquids during investigating the rheological properties is the dynamic test, in which the impacts of oscillating stress on the oil samples. There are two key properties known as loss modulus (G′) and storage modulus (G″) which are calculated. The loss modulus is known as viscous modulus and it indicates the energy dissipated as heat due to internal friction within the material during deformation, which is not recoverable. On the other hand, the storage modulus (G″) is known as elastic modulus and it reflects the elastic portion of the material's response. Moreover, represents the energy temporarily stored during deformation that can be recovered upon unloading.

**Fig. 12:**
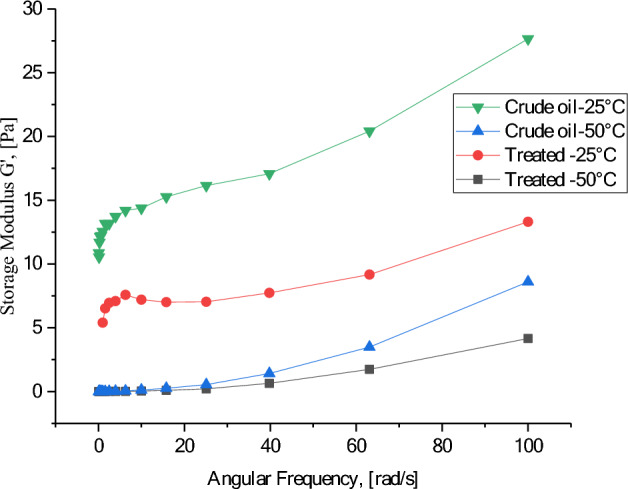
Storage modulus for crude oil before and after catalytic aquathermolysis using CSG2 at 25 and 50 °C.

**Fig. 13:**
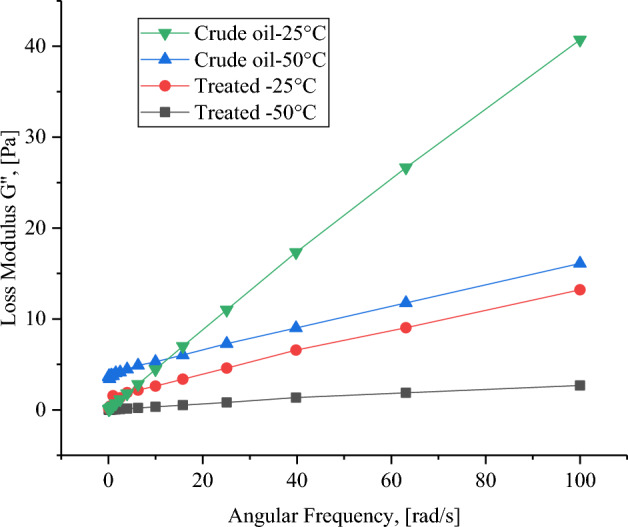
Loss modulus for crude oil before and after catalytic aquathermolysis using CSG2 at 25 and 50 °C.

Figures 12 and 13 show the frequency dependence of the storage modulus (G′) and loss modulus (G″) for the heavy crude oil before and after aquathermolysis with CSG2. It is obvious that the storage modulus increases with increasing angular frequency. This indicates that the oil become stiffer as the oscillation rate increases. In addition, the treated oil with CSG2 has a storage modulus lower than the untreated crude oil at both temperatures 25 and 50 °C. The heavy crude oil exhibits a nearly linear response across the investigated frequency range. Importantly, the loss modulus (G″) consistently exceeds the storage modulus (G′), indicating that the energy stored within the heavy crude oil is less than the energy dissipated. This suggests a more viscous liquid-like behavior than a solid-like one.

Table 6 compares the effectiveness of CSG1 and CSG2 against other published catalysts that were used in the catalytic aquathermolysis process. Notably, CSG2 achieved a significant 82% reduction in heavy crude oil viscosity. This catalyst holds promise for the petroleum industry due to its cost-effective preparation and the moderate temperature required for such high efficiency. The use of commercial-grade catalysts can improve the crude oil using aquathermolysis to a large extent, it has a negative effect represented by the synergetic effect of the ultrasonic and the catalyst^43^. However, the use of iron naphthenates in the catalytic aquathermolysis reduced the viscosity of heavy oil greatly it has a potential formation of stable emulsions. These stable emulsions can impede the interaction between the catalyst and the heavy oil, ultimately reducing the efficiency of the aquathermolysis process^44^.

**Table 6:** A comparison demonstrates the effect of the prepared catalysts and the previously published on the viscosity reduction during the catalytic aquathermolysis of heavy crude oil.

Oil source	Catalyst	Temperature, °C	Viscosity reduction, %	References
Niujuan heavy oil	Nickle oleate	220	65	^45^
Shengli oilfield	XAGD-2, Commercial grade	200	86	^43^
Heavy oil	Fe_3_O_4_	120	30	^46^
Residual oil	Iron naphthenate	340	75.91	^44^
Egyptian heavy oil	CSG1	225	62	This work
Egyptian heavy oil	CSG2	225	82	This work

## Conclusion

The synthesis and characterization of matrix polymer carboxyl methyl cellulose/silicate graphene oxide nanocomposites (CSG1 and CSG2) have demonstrated significant potential for enhancing the properties of Egyptian heavy oil. The catalytic performance of these nanocomposites was rigorously evaluated through a series of orthogonal and single-factor experiments. The study revealed that both CSG1 and CSG2 effectively reduce the viscosity of heavy crude oil, with CSG2 showing a more pronounced effect, achieving a viscosity reduction of up to 82%. SARA analysis showed that catalytic aquathermolysis increased lighter hydrocarbons while reducing heavier components. Resins dropped by 7.7% and 17.9%, and asphaltenes declined by 28.3% and 41.6% for CSG1 and CSG2, respectively. This indicates that CSG2 has a greater effect than CSG1. The process primarily targets asphaltene macromolecules, breaking them down into lighter, more desirable hydrocarbon fractions, thereby improving the flowability and transportability of heavy crude oil. Further investigations using FT-IR spectroscopy and gas chromatography confirmed the significant chemical changes in the treated oil, including the disappearance of specific peaks associated with heavy components and an increase in lighter hydrocarbons. These findings suggest that the catalytic aquathermolysis process effectively targets and degrades asphaltene macromolecules. Rheological studies demonstrated that treated crude oil exhibits lower storage modulus (G′) and higher loss modulus (G″) compared to untreated oil, indicating a more viscous liquid-like behavior. This change in rheological properties is beneficial for the transportation and processing of heavy crude oil. Consequently, the use of CSG2 nanocomposite in catalytic aquathermolysis has shown substantial improvements in reducing the viscosity and modifying the composition of heavy crude oil, thereby enhancing its flow properties and potential for efficient transport. These results underscore the effectiveness of these nanocomposites as catalysts for improving heavy oil recovery and processing. We can conclude that this study underscores CSG2's potential as a catalyst in catalytic aquathermolysis, offering valuable insights into its synthesis, characterization, and catalytic performance, thus advocating for its utilization in enhancing the economic viability of heavy oil projects.

## Data Availability

The raw data used and/or analysed during the current study is available from the corresponding author upon reasonable request.
